# Reporting of Telehealth-Delivered Dietary Intervention Trials in Chronic Disease: Systematic Review

**DOI:** 10.2196/jmir.8193

**Published:** 2017-12-11

**Authors:** Molly M Warner, Jaimon T Kelly, Dianne P Reidlinger, Tammy C Hoffmann, Katrina L Campbell

**Affiliations:** ^1^ Faculty of Health Sciences and Medicine Bond University Robina Australia; ^2^ Centre for Research in Evidence-Based Practice Faculty of Health Sciences and Medicine Bond University Robina Australia

**Keywords:** telemedicine, diet, chronic disease, behavior, review

## Abstract

**Background:**

Telehealth-delivered dietary interventions are effective for chronic disease management and are an emerging area of clinical practice. However, to apply interventions from the research setting in clinical practice, health professionals need details of each intervention component.

**Objective:**

The aim of this study was to evaluate the completeness of intervention reporting in published dietary chronic disease management trials that used telehealth delivery methods.

**Methods:**

Eligible randomized controlled trial publications were identified through a systematic review. The completeness of reporting of experimental and comparison interventions was assessed by two independent assessors using the Template for Intervention Description and Replication (TIDieR) checklist that consists of 12 items including intervention rationale, materials used, procedures, providers, delivery mode, location, when and how much intervention delivered, intervention tailoring, intervention modifications, and fidelity. Where reporting was incomplete, further information was sought from additional published material and through email correspondence with trial authors.

**Results:**

Within the 37 eligible trials, there were 49 experimental interventions and 37 comparison interventions. One trial reported every TIDieR item for their experimental intervention. No publications reported every item for the comparison intervention. For the experimental interventions, the most commonly reported items were location (96%), mode of delivery (98%), and rationale for the essential intervention elements (96%). Least reported items for experimental interventions were modifications (2%) and intervention material descriptions (39%) and where to access them (20%). Of the 37 authors, 14 responded with further information, and 8 could not be contacted.

**Conclusions:**

Many details of the experimental and comparison interventions in telehealth-delivered dietary chronic disease management trials are incompletely reported. This prevents accurate interpretation of trial results and implementation of effective interventions in clinical practice.

## Introduction

Telehealth is an effective mode for delivering dietary interventions [1,2]. There is a strong relationship between dietary quality and the prevention and management of chronic diseases [3] including diabetes [4], cardiovascular disease [5], and obesity [6]. Telehealth-delivered dietary interventions have been shown to significantly improve blood pressure, cholesterol, triglycerides, body weight, and waist circumference in people with chronic diseases [2]. There are a multitude of barriers to face-to-face dietary interventions, including nonattendance to clinics, transport problems, inflexible hours, long wait times, and cost for both the patient and the practitioner [1,2,7]. These barriers can be addressed by adopting telehealth, which has been accepted by participants in dietary behavior change [2,8] and chronic disease management [9-11] studies. Although its use is promising, telehealth is a widely used term, and its emerging use in clinical practice is broad and varied [2,9,10,12-14]. Telehealth methods including mobile health and electronic health, may involve delivery of health care via telephone, SMS text message (short message service, SMS), email, video, website, and other remote devices. These devices can be used for one-on-one consultations, store-and-forward education, behavior change reminders, and remote monitoring and feedback. There remain a number of challenges for introducing telehealth into health care systems, such as inconsistent terminology, evolving telehealth technologies, and limited public and private health funding for implementation into standard care [12]. Developing a strong evidence-base for the use of telehealth will help to better understand how to overcome such challenges.

To implement effective telehealth interventions, practitioners need to know what telehealth is and how it is used. Translating knowledge from trials into clinical practice is crucial for improving health care and chronic disease management. However, this translation is challenged when trials are poorly reported and provide insufficient detail for implementing evidence-based interventions in practice [15-18].

In addition to the complexity of telehealth delivery, dietary behavior change interventions also have many layers of complexity in terms of the number of dietary factors targeted; the need for comprehensive individualized behavior change techniques; interrelated lifestyle behaviors; and the influence of social and environmental circumstances, attitudes, and skill levels [19,20]. Complex nonpharmacological interventions have been recently shown to be poorly reported [21-24]. To our knowledge, no previous studies have examined the reporting of interventions in dietary or telehealth-delivered trials. In addition to the complete reporting of experimental intervention components, it is important that comparison or control interventions are completely described to allow accurate interpretation and evaluation of effect size within and across trials.

This review aimed to evaluate the completeness of intervention reporting of experimental and comparison interventions in published dietary chronic disease management trials that used telehealth delivery methods.

## Methods

### Study Design

This study is a secondary analysis of the articles identified in a systematic review that examined the effectiveness of telehealth-delivered dietary interventions in chronic disease [2].

### Search Strategy

Eligible studies were identified from a systematic review of randomized controlled trials (RCTs) using telehealth methods to deliver multifactorial dietary interventions in adults with chronic disease, conducted by our team [2]. A literature search was performed across multiple electronic databases (MEDLINE, EMBASE, CINAHL, and PsychINFO) up to November 2015, as detailed previously [25]. A multi-step search approach was taken to retrieve relevant trial publications for this study using forward and backward citation searching; expert correspondence; and searching conference abstracts, theses, dissertations, and clinical trial registries to identify ongoing trials. Two researchers (JK and MW) independently screened the search articles, and disagreements were resolved by discussion.

### Trial Publication Selection

Trial publications were included in this review if they were RCTs, cluster RCTs, or quasi-RCTs conducted in adults (>18 years of age) with at least one diet-related chronic disease. Experimental interventions were required to include two or more dietary components (eg, vegetables and whole grains). Half of the total intervention contact hours or interaction contacts was required to be delivered by telehealth and must have been developed or delivered by a qualified health professional. This study includes all telehealth-delivered dietary interventions, regardless of reporting of dietary outcomes. Studies analyzed in this study met the inclusion criteria as outlined in the systematic review protocol [25]. The original review included 25 studies with diet outcome data; however, this current reanalysis includes an additional 12 studies without diet outcome data, which otherwise met the inclusion criteria for this review. All 37 studies were therefore analyzed for completeness of reporting of the intervention, regardless of the reporting of outcome data.

### Assessment of Trial Reporting

To appraise the completeness of reporting of telehealth-delivered dietary interventions, the Template for Intervention Description and Replication (TIDieR) checklist and guide [18] was used. The 12-item TIDieR checklist is an extension of item 5 of the consolidated standards of reporting trials (CONSORT) 2010 statement [26] and item 11 of the Standard Protocol Items: Recommendations for Interventional Trials checklist [27].

The completeness of reporting of experimental and comparison interventions in each trial was recorded on a data extraction form (Multimedia Appendix 1) based on the TIDieR checklist [18]. If trials had more than one experimental intervention group, the interventions were assessed separately. Two researchers (MW and JK) independently assessed each trial and discussed differences in the rating of TIDieR items. There was an 88% agreement between the two reviewers before the initial discussion. After reappraisal and further discussion, less than 1% of items appraised were conflicting, which were then resolved with discussion to reach a consensus. If consensus could not be achieved, a third researcher (TH) was available to resolve any conflicts.

### Procedure for Attaining Additional Intervention Information

Reference lists, clinical trial registration records, available trial protocols, and trial authors’ research profiles were screened to determine whether additional written information about each trial’s intervention was publicly available. Information obtained from these sources was considered, and checklist items were rescored as *complete from additional sources* where relevant. For items remaining incomplete, attempts were made to contact trial authors by emailing them questions specifically related to the incomplete items for the experimental interventions. Where corresponding author email addresses were unavailable, attempts were made to search for alternate email addresses and contact other authors via email. Authors were sent up to three email reminders, each approximately 3 weeks apart. Author responses were used to rescore the TIDieR checklist.

### Data Analysis

Data were analyzed using descriptive statistics (number and percentages) in Excel 2010 (Microsoft).

## Results

### Characteristics of Included Trials

**Figure 1:**
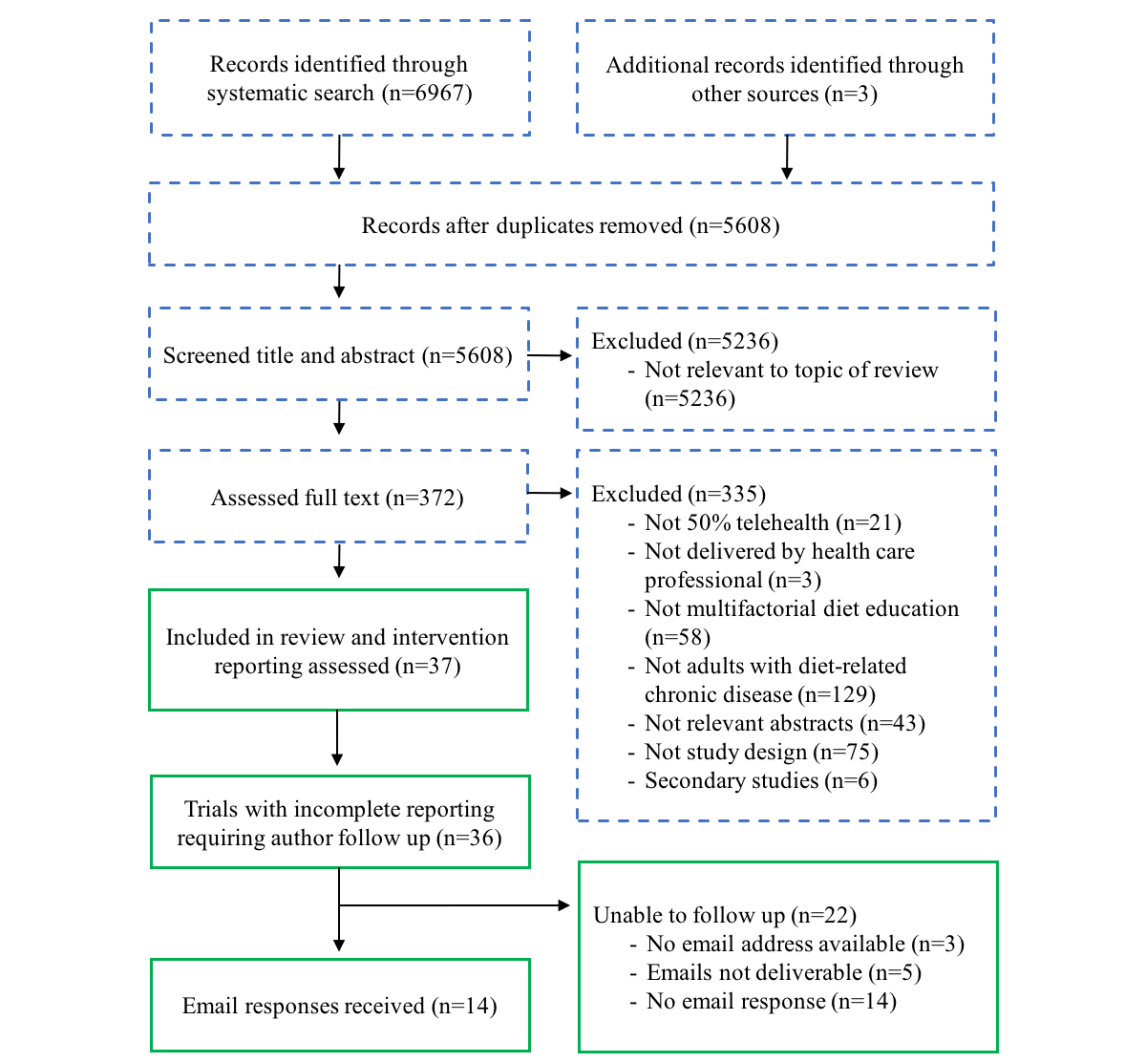
Flow of the trial publication selection and author contact process (blue or dashed boxes represent the steps taken as part of the existing systematic review; green or line boxes were steps taken for this study).

A total of 37 trials were included (Figure 1) [2], of which 49 were experimental interventions and 37 were comparison interventions. Of the 37 trials, 29 evaluated one experimental intervention [7,8,14,28-53], 4 trials evaluated two experimental interventions [54-57], and 4 trials evaluated three experimental interventions [58-61]. Trials were published from 1981 and 2016 and conducted in patients with cardiovascular disease or heart failure (n=13) [7,8,29,31,36-40,42,56,57,62], hypertension (n=11) [14,32-34,43,44,54-56,59,60], diabetes (n=10) [14,28,30,35,45-50,56,61], kidney disease (n=3) [51,52,58], and obesity (n=3) [32,53,57]. The majority of trials involved face-to-face interaction between intervention providers and participants before the telehealth component of the intervention.

**Figure 2:**
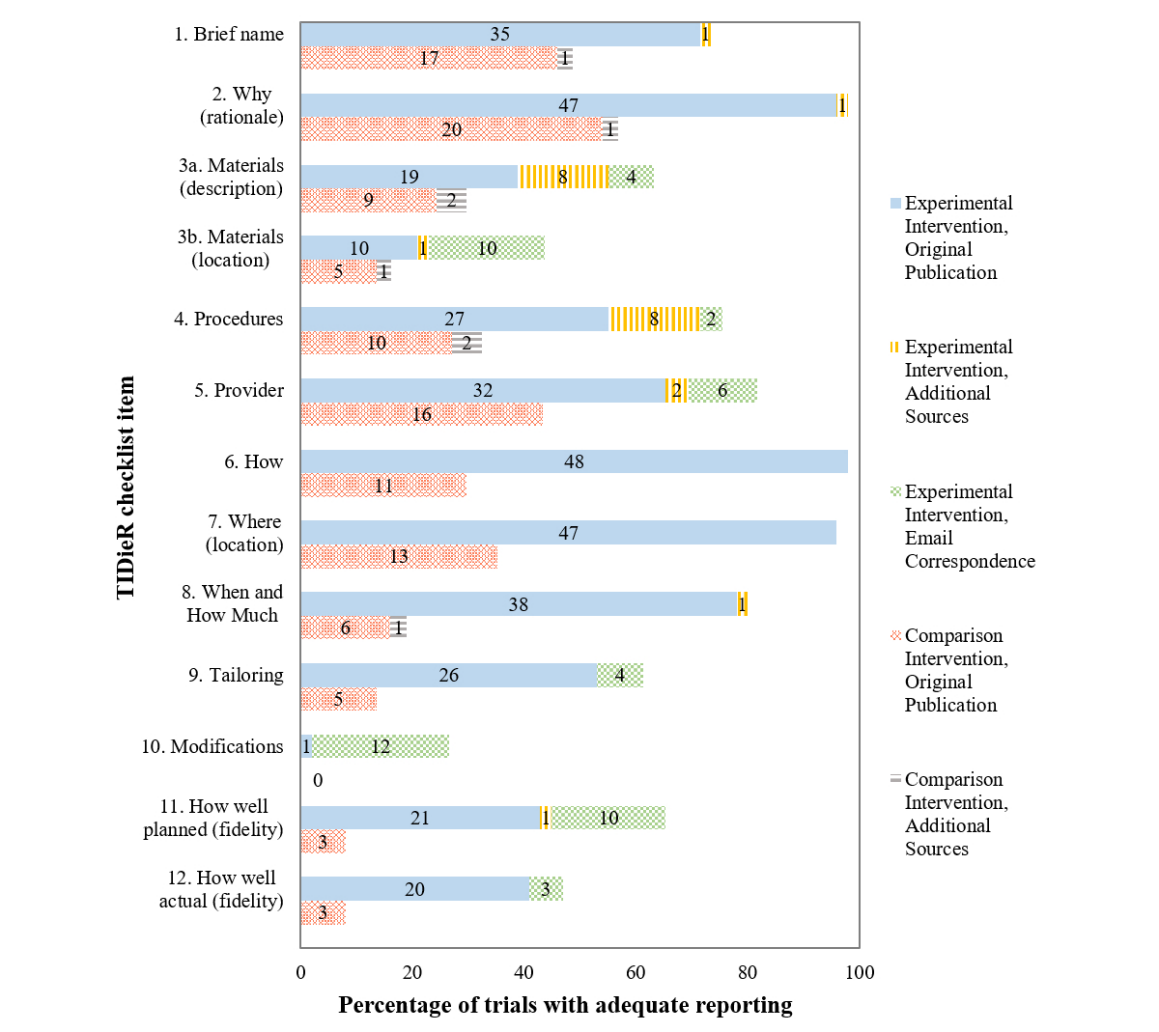
Items with complete reporting across the 12 Template for Intervention Description and Replication (TIDieR) checklist items in 49 experimental and 37 comparison interventions of dietary trials delivered by telehealth. Numbers in bars represent the number of interventions rated as complete.

Most interventions (70%) used the telephone as the telehealth delivery method, others used short message service (SMS), the Internet, video, videoconferencing, and a mix of telehealth methods. Figure 2 shows the percentage and number of experimental and comparison interventions that completely reported each TIDieR checklist item in the original trial publication, in additional sources of published information, and after email correspondence with authors.

### Reporting of Experimental Interventions

Nearly all (98%, 48/49) experimental interventions were incompletely reported in their original publication. Only one publication [8] completely reported every checklist item. Items that were commonly reported included how or the mode of delivery (item 6), rationale (item 2), and location (item 7) of the intervention. Items with the poorest reporting were materials (items 3a-b), modifications to the intervention (item 10), and fidelity (items 11-12). Intervention materials such as training materials, questionnaires, handbooks, leaflets, videoconferencing units, short SMS text messages, or websites were used in all trials. Interventions with incomplete reporting of procedures (item 4) commonly missed details required for replicating the dietary advice provided to participants.

### Reporting of Comparison Interventions

The majority (78%, 29/37) of comparison interventions were described as “usual care,” whereas others (22%, 8/37) were “control interventions” with less intensive procedures (eg, education sessions without telephone or email follow-up, resources, or extra video education). The most commonly reported items were rationale (item 2), brief name (item 1), and provider (item 5). The least reported items were modifications (item 10), fidelity (items 11-12), materials (items 3a-b), tailoring (item 9), and when and how much of the comparison intervention was provided (item 8). More comparison interventions had intervention details incompletely reported than experimental interventions.

### Searching Additional Sources and Contacting Authors for Intervention Information

Although descriptions of the materials used in the experimental intervention were poorly reported in the original publications (39%, 19/49), details were provided in additional sources of information (16%, 8/49) and by contacting authors through email (8%, 4/49). The locations of the materials used in the experimental intervention were further reported in email correspondence with authors (20%, 10/49). Searching additional sources of published information was time-consuming and only satisfied an additional 3% and 2% of checklist items for experimental and comparison interventions, respectively. Likewise, attaining information through email required 40 reminder emails to be sent; only 39% (14/36) of authors replied with further information, and author responses were up to 8 weeks after the initial email.

## Discussion

### Principal Findings

This study aimed to evaluate the completeness of reporting of experimental and comparison interventions in dietary chronic disease management trials that used telehealth delivery methods. The key finding was that only one experimental intervention (2%) and no comparison interventions were reported in enough detail to satisfy every TIDieR checklist item. This finding illustrates a major deficit in the reporting of information that is required for health professionals to accurately replicate dietary interventions.

Findings from this study are consistent with other evaluations of the completeness of reporting of nonpharmacological interventions that have found poor reporting across trials of physiotherapy, occupational therapy, smoking cessation, and cardiac and stroke rehabilitation interventions [21-23,62-64]. Reporting of the experimental intervention rationale, mode of delivery, and location or setting was complete in most trials, which is consistent with findings of other studies [21-23]. Although details of intervention providers were not well described in the included publications (65%, 32/49 experimental interventions; 43%, 16/37 comparison interventions), we found a greater proportion of complete reporting compared with previous studies; whereby, details on intervention providers were reported in 59% of original cardiac rehabilitation intervention publications [22] and 38% interventions for upper limb therapies in cerebral palsy [23]. Reporting of the delivery mode, location or setting, and provider details may have been inflated in this study because of the restrictive and predefined inclusion criteria for selecting relevant trials.

Accurate interpretation of intervention effects is limited when the dose and frequency of dietary support or education in each of the experimental and comparison interventions is unknown. The amount (dose) of contact, for example, has been shown to be positively associated with sustained dietary behavior change [1]. Reporting of comparison intervention details, including the dose and frequency of intervention delivery, is necessary for accurate interpretation and evaluation of treatment effect size within and across trials.

Most comparison interventions (78%, 29/37) were briefly described as simply “usual care.” This is of concern because usual care is likely to differ for participants within and between trials because of a multitude of determinants including the health professional(s) and other personnel involved and the country’s health care system [23]. The completeness of reporting of comparison interventions in randomized trials has been explored previously; whereby, less than 40% of publications completely report the procedures, materials used, mode of delivery, tailoring, modifications, and planned and actual fidelity of comparison interventions [23,64]. Comparison interventions should be reported more completely to allow health professionals to make a clinical judgment on the additional benefit of an experimental intervention.

Trial publications with complete descriptions of physical and informational materials allow readers to use the materials of effective interventions in practice. This study found that descriptions of materials (39%, 19/49 experimental interventions) and where to access materials (21%, 10/49 experimental interventions) were poor, which is similar to previous findings [23,64]. If authors are unable to describe the materials completely in the main publication, they need to specify where further information about or the actual information materials can be found so that all elements of effective telehealth interventions can be used in practice.

Multifactorial dietary behavior change trials, regardless of their mode of delivery, are complex in comparison with trials of simple or single interventions. This is partly because of internal and external influencing factors including social and environmental circumstances, attitudes, and skill levels [19,20]. Tailoring chronic disease management strategies to support individualized dietary and lifestyle behavior change is particularly important. Tailoring of experimental interventions to trial participants was reported in only 53% (26/49) of trial publications. Many interventions were *tailored to each individual*, yet few trials reported the rationale, guides, variables, or constructs used for participant assessment, decision points, or actions for tailoring (eg, questionnaire to determine adherence to diet at a specific time point) [18]. Completely describing tailoring is challenging; however, detailed descriptions help readers to distinguish between intentional tailoring and poor fidelity [65]. As consistent taxonomy for behavior change techniques are further developed [16], reporting of tailoring for behavior change interventions will hopefully become more widespread.

Assessing fidelity in dietary behavior change trials is similarly complex in comparison with simple trials [18,65]. Intervention fidelity encompasses aspects such as the intervention design, delivery and receipt, and how well participants are able to use learned skills outside of formal intervention sessions [66]. Reporting of intervention fidelity is required for readers to accurately interpret reliability and validity, as well as optimize the efficacy of future interventions and clinical practice. Similar to findings in this study, fidelity of complex behavior change has previously found to be poorly reported [67]. For example, 87% (146/168) of behavioral pediatric obesity intervention trials reported less than half of assessed fidelity components.

Although word or page limits in peer-reviewed journals may be one of the restrictions perceived by authors as a barrier to fully describing interventions [68], Web-based supplementary materials and publishing of detailed trial protocols may assist in overcoming restrictions [19,23]. The incomplete intervention reporting in our sample of studies may have occurred for a number of reasons including lack of awareness by trial authors about what constitutes a complete intervention description and the importance of it; no requirement to adhere to TIDieR checklist by most journals in which telehealth-delivered dietary trials are published; and publication of studies before release of the TIDieR checklist in 2014, although the CONSORT extension for nonpharmacological interventions was published in 2008 and contains some expanded guidance for reporting interventions.

This study is the first to evaluate the completeness of intervention reporting in trials of dietary intervention delivered by telehealth methods. Strengths of this study include the thorough evaluation by two independent reviewers, of intervention reporting including evaluation of additional sources of published information, and email correspondence with authors. Although the TIDieR checklist is extensive, it does not directly specify all variables that may influence the outcome of the intended intervention, such as personal attributes of the person delivering the intervention. The majority of the included trials involved physical activity and lifestyle components, as well as dietary behavior change components. As the scope of this study was limited to telehealth-delivered dietary interventions, conclusions on the reporting of other telehealth interventions cannot be drawn. This study highlights that trials of complex interventions need to report each component of chronic disease management completely for accurate evaluation and replication of components of the trial, or the trial as a whole.

### Conclusions

Intervention details of dietary trials delivered by telehealth methods are not adequately reported, limiting their replication in research and clinical practice. The least reported items of the experimental intervention were descriptions and locations of the physical and informational materials used. Reporting of comparison intervention details needs to be more complete to allow evaluation of the additional benefit of experimental interventions. Inadequate reporting of trials prevents closure of the translational gap between research trials and clinical practice, thereby limiting the potential for health care professionals to implement effective interventions to assist people with managing their chronic disease. Our findings confirm the pressing need for authors, editors, and reviewers to use the TIDieR checklist to ensure complete reporting of published dietetic trials.

## Appendix

Multimedia Appendix 1 Template for Intervention Description and Replication (TIDieR) checklist and examples of adequate reporting in included trial publications.

## Abbreviations

CONSORT: consolidated standards of reporting trials
RCT: randomized controlled trial
SMS: short message service
TIDieR: Template for Intervention Description and Replication
