# Supplementary material for: Reporting of Telehealth-Delivered Dietary Intervention Trials in Chronic Disease: Systematic Review
Source: J Med Internet Res. 2017 Dec 11;19(12):e410. doi: 10.2196/jmir.8193 (PMC5742660; doi:10.2196/jmir.8193)
Supplement: Multimedia Appendix 1 [file jmir_v19i12e410_app1.pdf]

Multimedia Appendix 1. Template for Intervention Description and Replication (TIDieR) checklist (adapted from reference [1]) and examples of adequate reporting in included trial publications.

**Item**	**Item description**	**Example of adequate reporting**
1. Brief name	A name or a phrase which describes the intervention	"*Trial to Examine Text Message-Based mHealth in Emergency Department Patients with Diabetes (TExT-MED).*" [2]
2. Why	Describes the rationale, theory, or goal of the elements essential to the intervention	*"To encourage lifestyle change, the intervention was based on social cognitive theory and the key mediator of self-efficacy. Perceived self-efficacy is the extent to which people believe they can exercise control over their health behaviors. With higher levels of self-efficacy, individuals can self-regulate their behavior by setting goals, creating incentives, and enlisting social support from others to maintain their motivation..."* [3]
3. What: materials		
a. Description	Describes any physical or informational materials provided to participants used in intervention delivery or in training of intervention provider	Example text message used in the TEXT ME randomized clinical trial: *"did you know 90% of people don't eat the recommended daily intake of vegetables (5 serves a day)?"* [4]
b. Location	Provides a link, appendix, or reference to where the materials can be located	"*Participants should be able to perform blood glucose self-testing and -injection of medication, and input to the website [website address].*" [5]
4. What: procedures	Describes each of the procedures, activities, and processes used in the intervention,	"*Participants were guided through a series of steps beginning with an assessment of their [coronary heart disease] risk factor profile at the commencement of the program...participants were*

	including any enabling or support activities, and the specific type of dietary education provided, including which nutrient(s) or food(s) were targeted or which strategies were implemented.	*encouraged to set Specific, Measurable, Achievable, Realistic and Timely (SMART) goals...Barriers and enablers were identified, confidence was assessed and problem-solving was discussed if necessary. Subsequent sessions were structured as follows...Risk factor advice was consistent with the National Heart Foundation of Australia's guidelines with the goal of achieving the following...five servings of vegetables and two servings or fruit per day, <30% and 10% of calories from total fat and saturated fats, respectively, <2,300 mg sodium per day and <300 mg dietary cholesterol per day; <2 and <1 standard alcoholic drinks per day for men and women, respectively...*" [6]
5. Who provided	Describes the intervention provider and their expertise, background, and any specific training given	"*Study dietitians worked for [the research institute] before and during the study and were experienced with caring for patients with diabetes; obesity (particularly morbid obesity pre- and post-bypass surgery); and chronic diseases. Dietitians received three half-day study training sessions, which included review of guidelines for hypertension care and primary prevention of CVD, including stepped medication guidelines for elevated BP and lipid lowering, and training on the research protocol. They were also trained in and practiced motivational interviewing and cognitive behavioral skills training techniques, which they used in counseling participants.*" [7]
6. How	Describes the modes of delivery (eg, telephone, mobile, and Internet) of the intervention and whether it was provided	*"[Patients] completed a face-to-face baseline assessment in hospital, a clinic, or home setting within 4 weeks of hospital discharge...A theoretically framed comprehensive program of evidence-based [community resource] guidelines*

	individually or in a group	*was delivered by text message and a supporting website over 24 weeks.”* [3]
7. Where	Describes the type(s) of location(s) where the intervention occurred, including any necessary infrastructure or relevant features	*“...contacted by telephone at home.”* [8]
8. When and how much	Describes the dose of dietary education used in the intervention (eg, every call or 2 messages per week) and the frequency of sessions or contact hours for telehealth intervention	*“Participants in the DASH-Plus group received a single, 1-hour, in-person, one-on-one session with the study coach who delivered a dietitian-developed module on adoption of the DASH diet. This was followed by weekly 15-minute calls with the participant during the 8-week study period.”* [9]
9. Tailoring	Describes the what, why, when, and how of intervention titration, personalization, or progression	*“During each call...the stage of change for adherence to diet...was assessed separately using the validated stage of change questions and tailored counseling based on this assessment. The stages of change were...Patients were considered adherent to diet if they reported eating the appropriate diet for hypertension...≥6 days per wk... The intervention was tailored to target personal barriers and brainstorm solutions...Patients received tailored counseling for each target behavior based on their current stage of change. [Stage Matched Intervention] used the processes of change using the cognitive and behavioral activities found to be most effective for each stage and incorporated decisional balance and self-efficacy. For the decisional balance, the pros and cons of each*

		behavior were elicited, and the counselor explored why each pro endorsed was important to the participant. For each con, alternatives were explored using problem-solving methods. Similarly, for self-efficacy, the counselor worked with the participant to enhance confidence in ability to adhere." [10]
10. Modifications	Describes any modifications to the intervention during the course of the study	*"No changes were made to the intervention content of delivery during the study period"* [3]
11. How well: planned	Describes strategies used to maintain or improve fidelity (how and by whom)—that is, how did the authors plan to assess how consistent the intervention was delivered (eg, audit of phone calls or recording number of text replies)	"*Fidelity of intervention delivery was monitored via feedback to counselors following randomly recorded telephone calls and fortnightly clinical supervision meetings."* [11]
12. How well: actual	Describes the extent to which the intervention was delivered as planned (if adherence or fidelity was assessed)—that is, are the results of how consistent the intervention was delivered reported? (eg, average call durations or number call attempts or number of text replies)	*"Respectively, the completion of ≥75% of scheduled calls was achieved by 36.4% (55 of 151) of telephone counseling group participants or 57.6% (53 of 92) of telephone counseling participants who had not withdrawn from the intervention or the study. The mean (±SD) duration of intervention calls was 24.6 ± 10.6 min."* [11]

References

1. Hoffmann TC, Glasziou PP, Boutron I, Milne R, Perera R, Moher D, Altman DG, Barbour V, Macdonald H, Johnston M, Lamb SE, Dixon-Woods M, McCulloch P, Wyatt JC, Chan AW, Michie S. Better reporting of interventions: Template for intervention description and replication (tidier) checklist and guide. BMJ. 2014 Mar 07;348:g1687. PMID: 24609605. doi: 10.1136/bmj.g1687.

2. Arora S, Peters AL, Burner E, Lam CN, Menchine M. Trial to examine text message-based mhealth in emergency department patients with diabetes (text-med): A randomized controlled trial. Ann Emerg Med. 2014 Jun;63(6):745-54 e6. PMID: 24225332. doi: 10.1016/j.annemergmed.2013.10.012.

3. Pfaeffli Dale L, Whittaker R, Jiang Y, Stewart R, Rolleston A, Maddison R. Text message and internet support for coronary heart disease self-management: Results from the text4heart randomized controlled trial. J Med Internet Res. 2015 Oct 21;17(10):e237. PMID: 26490012. doi: 10.2196/jmir.4944

4. Chow CK, Redfern J, Hillis GS, Thakkar J, Santo K, Hackett ML, Jan S, Graves N, de Keizer L, Barry T, Bompoint S, Stepien S, Whittaker R, Rodgers A, Thiagalingam A. Effect of lifestyle-focused text messaging on risk factor modification in patients with coronary heart disease: A randomized clinical trial. JAMA. 2015 Sep 22-29;314(12):1255-63. PMID: 26393848. doi: 10.1001/jama.2015.10945.

5. Hee-Sung K. Impact of web-based nurse's education on glycosylated haemoglobin in type 2 diabetic patients. J Clin Nurs. 2007 Jul;16(7):1361-6. PMID: 17584355. doi: 10.1111/j.1365-2702.2007.01506.x.

6. Hawkes AL, Patrao TA, Atherton J, Ware RS, Taylor CB, O'Neil A, Foreman R, Oldenburg BF. Effect of a telephone-delivered coronary heart disease secondary prevention program (proactive heart) on quality of life and health behaviours: Primary outcomes of a randomised controlled trial. International journal of behavioral medicine. 2013 Sep;20(3):413-24. PMID: 23012159. doi: 10.1007/s12529-012-9250-5.

7. Green BB, Anderson ML, Cook AJ, Catz S, Fishman PA, McClure JB, Reid RJ. E-care for heart wellness: A feasibility trial to decrease blood pressure and cardiovascular risk. Am J Prev Med. 2014 Apr;46(4):368-77. PMID: 24650839. doi: 10.1016/j.amepre.2013.11.009.

8. Chiu CW, Wong FK. Effects of 8 weeks sustained follow-up after a nurse consultation on hypertension: A randomised trial. Int J Nurs Stud. 2010 Nov;47(11):1374-82. PMID: 20413121. doi: 10.1016/j.ijnurstu.2010.03.018.

9. Miller ER, 3rd, Cooper LA, Carson KA, Wang NY, Appel LJ, Gayles D, Charleston J, White K, You N, Weng Y, Martin-Daniels M, Bates-Hopkins B, Robb I, Franz WK, Brown EL, Halbert JP, Albert MC, Dalcin AT, Yeh HC. A dietary intervention in urban african americans: Results of the "five plus nuts and beans" randomized trial. Am J Prev Med. 2016 Jan;50(1):87-95. PMID: 26321012. doi: 10.1016/j.amepre.2015.06.010.

10. Friedberg JP, Rodriguez MA, Watsula ME, Lin I, Wylie-Rosett J, Allegrante JP, Lipsitz SR, Natarajan S. Effectiveness of a tailored behavioral intervention to improve hypertension control: Primary outcomes of a randomized controlled trial. Hypertension. 2015 Feb;65(2):440-6. PMID: 25403606. doi: 10.1161/HYPERTENSIONAHA.114.03483.

11. Eakin EG, Winkler EA, Dunstan DW, Healy GN, Owen N, Marshall AM, Graves N, Reeves MM. Living well with diabetes: 24-month outcomes from a randomized trial of telephone-delivered weight loss and physical activity intervention to improve glycemic control. Diabetes Care. 2014 Aug;37(8):2177-85. PMID: 24658390. doi: 10.2337/dc13-2427.
